# Supplementary material for: A sensitive mNeonGreen reporter system to measure transcriptional dynamics in Drosophila development
Source: Commun Biol. 2020 Nov 12;3:663. doi: 10.1038/s42003-020-01375-5 (PMC7665215; doi:10.1038/s42003-020-01375-5)
Supplement: Supplementary file 1 — Supplementary Information [file 42003_2020_1375_MOESM1_ESM.pdf]

# Supporting Information for:

# A sensitive mNeonGreen reporter system to measure transcriptional dynamics in *Drosophila* development

Stefano Ceolin[1], Monika Hanf[1], Marta Bozek[1], Andrea Ennio Storti[1], Nicolas Gompel[2], Ulrich Unnerstall[1], Christophe Jung[*,1] and Ulrike Gaul[1]

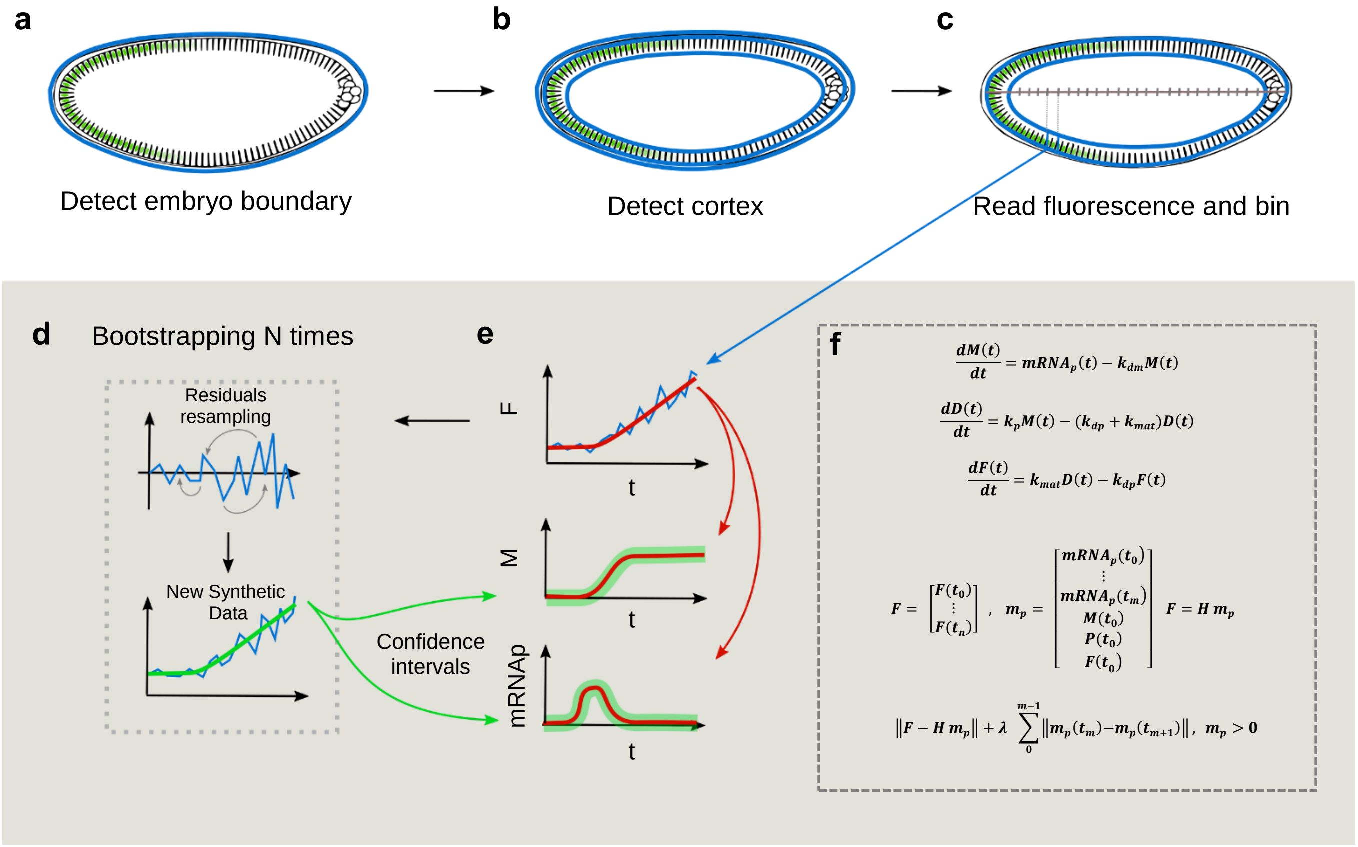


**Supplementary Figure 1. Data analysis pipeline for the mNeonGreen reporter. a-b-c** Overview of the image analysis pipeline: in order to read out the fluorescence of the reporter we first detect the embryo boundary. From the boundary we then grow two contours which delimit the cortical region of embryo. We read out the fluorescence in this region and bin the data based on the position along the AP axis of the embryo, using bins of 2% of embryo length. **d-e-f** Sketch of the mRNA reconstruction analysis. The time course of the signal in each bin is analyzed separately by fitting the model expressed by equations in **f** to the fluorescence data. The model is fitted to the data minimizing a regularized least squares. After the first fit of the data we apply a bootstrapping algorithm in **d** based on the resampling of the residuals. The residuals are reshuffled and added again to the fluorescence time course predicted by the model to obtain a new synthetic dataset. This new dataset is re-analyzed and the bootstrapping procedure is repeated N times to construct confidence intervals around the predicted mRNA and mRNA production levels. The reshuffling of the residuals takes into account that the residuals are uncorrelated but their amplitude depend on the signal intensity.

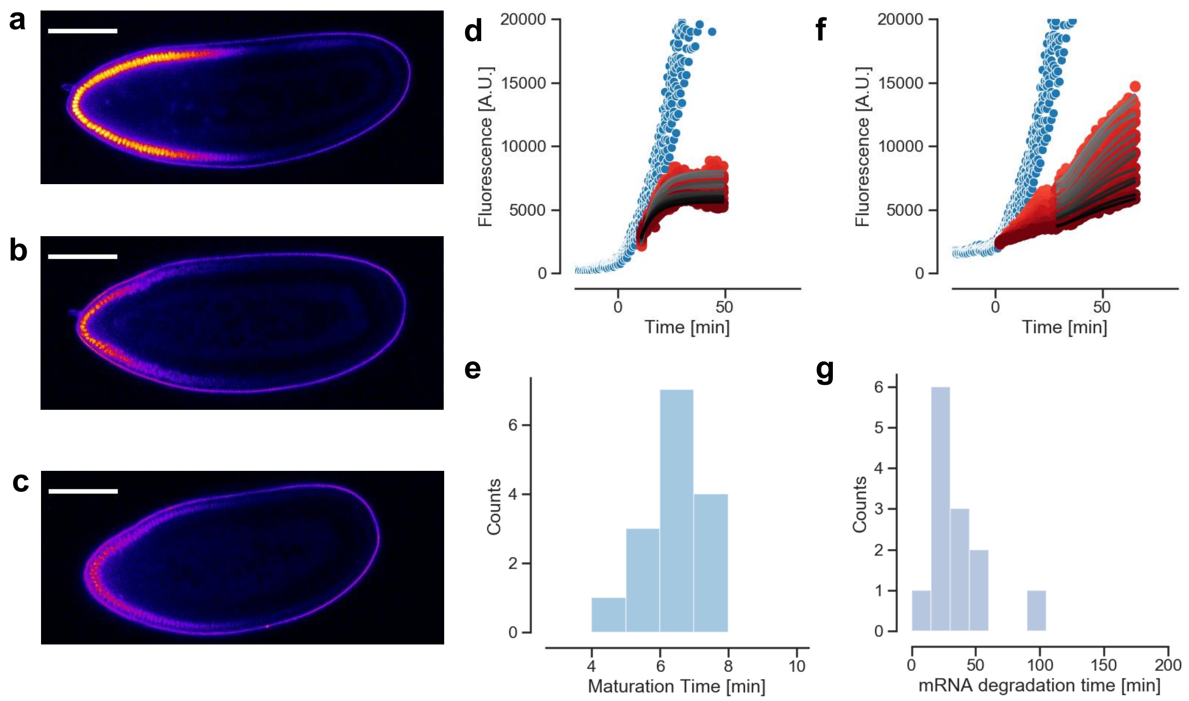


**Supplementary Figure 2. mNeonGreen reporter calibration.** Calibration experiments for the mNeon expression reporter. **a-b-c** Embryos have been injected with either water (**a**), the translation inhibitor cycloheximide (**b**) or the transcription inhibitor alpha-amanitin (**c**). Fluorescence from the mNeonGreen reporter has been monitored using confocal microscopy. (Scale bars 100μm). **d** Time course of the fluorescence signal at different positions in a cycloheximide injected embryo (in red) and a water injected embryo (in blue). Time series data from different positions in a cycloheximide injected embryo are modelled with exponential curves characterized by a maturation time t, which is defined as the inverse of the maturation rate (see **METHODS** for details about the fitting). **e** Estimates of the maturation time. The average maturation time over different positions is 6.58 minutes. **f** Time course of the signal at different positions in an alpha-amanitin injected embryo (in red) and a water injected embryo (in blue) The time courses of fluorescence at different positions in a alpha-amanitin injected embryo are modelled to infer the degradation rate of the reporter mRNA (See **METHODS** for details about the fitting). **g** Estimates of the reporter mRNA lifetime. The average reporter mRNA lifetime over different positions is 35 minutes.

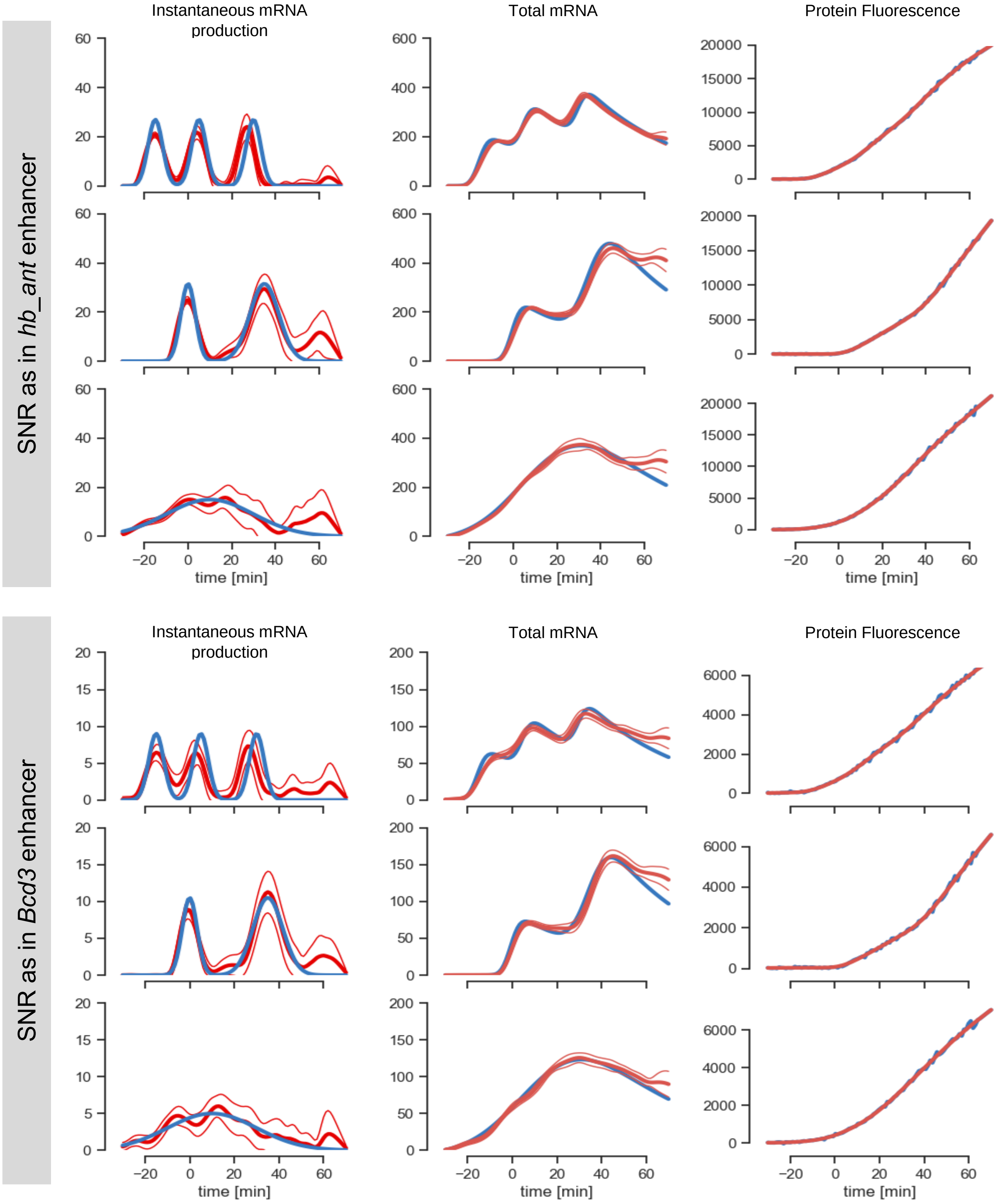
SNR as in hb_ant enhancer
Instantaneous mRNA production
Total mRNA
Protein Fluorescence
time [min]
SNR as in Bcd3 enhancer
Instantaneous mRNA production
Total mRNA
Protein Fluorescence
time [min]

**Supplementary Figure 3. Robustness tests of mRNA reconstruction analysis on simulated data.** Protein fluorescence data have been simulated starting from the time courses of mRNA production (leftmost column, in blue). Similar noise levels as those observed in our own experimental data have been added to the simulated fluorescence signal (rightmost column, in blue). The mRNA reconstruction algorithm is then used to reconstruct the total amount of mRNA and the rate of mRNA production rates by fitting the noisy fluorescence data. The result of the reconstruction and corresponding confidence intervals are plotted in red in each panel. The reconstruction algorithm is able to accurately capture the dynamics of mRNA production both in the case of high and low signal to noise levels. Generally, the reconstruction of instantaneous mRNA production is noisier than the total mRNA level, since rapid fluctuations in the rate of mRNA production are smoothed at the protein level. Note that the reconstruction is not reliable towards the end of the time series; this is due to the fact that the reconstruction of mRNA production at a given time is dependent only on the protein fluorescence at later times. At the end of the time series, there are simply fewer data points, leading to uncertainty in the reconstruction.

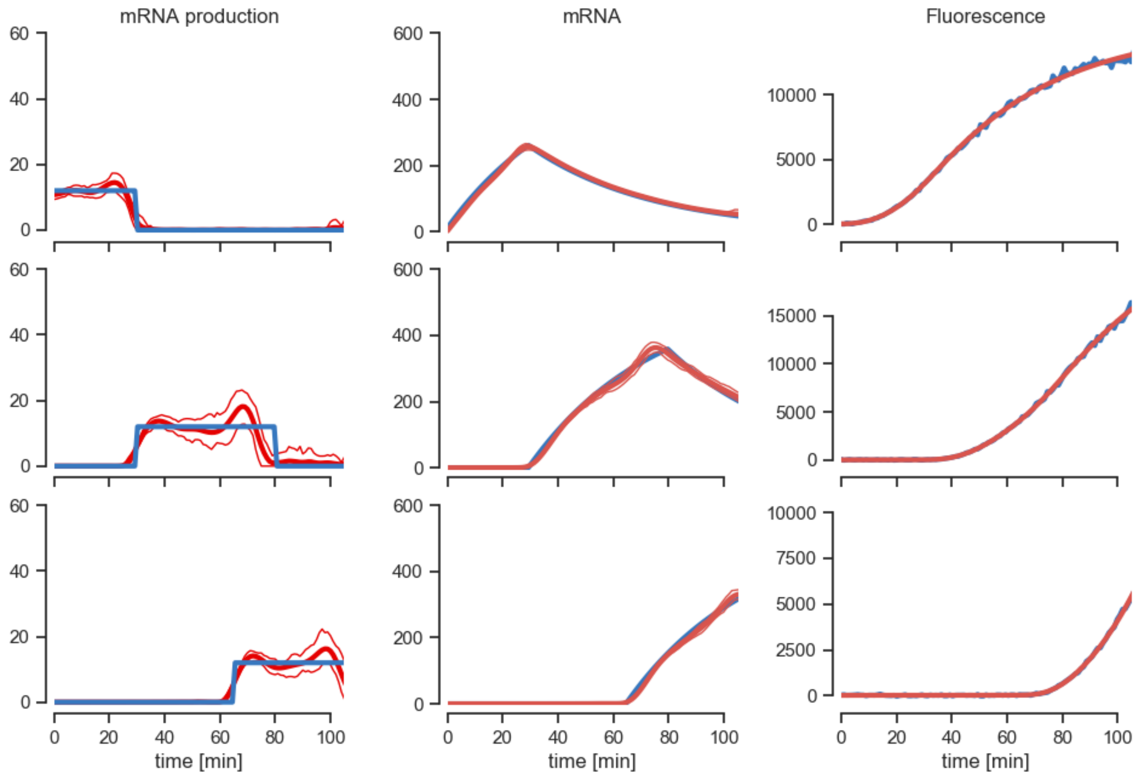


**Supplementary Figure 4. The mRNA reconstruction analysis can detect both enhancer activation and deactivation.** Protein fluorescence data were simulated starting from the time courses of mRNA production (leftmost column, in blue). Similar noise levels as those observed in our own experimental data have been added to the simulated fluorescence signal (rightmost column, in blue). The mRNA reconstruction algorithm is then used to reconstruct the total amount of mRNA and the rate of mRNA production rates by fitting the noisy fluorescence data. The result of the reconstruction and corresponding confidence intervals are plotted in red in each panel. We considered three scenarios, in which the enhancer is either already active at the beginning of the measurement (first row), only active in a limited time window (second row), or gets activated at some point during the measurement and remains active until the end of the experiment (last row). In all cases the algorithm was able to accurately detect the dynamics, both at the level of instantaneous mRNA production rate and for cumulative mRNA production.

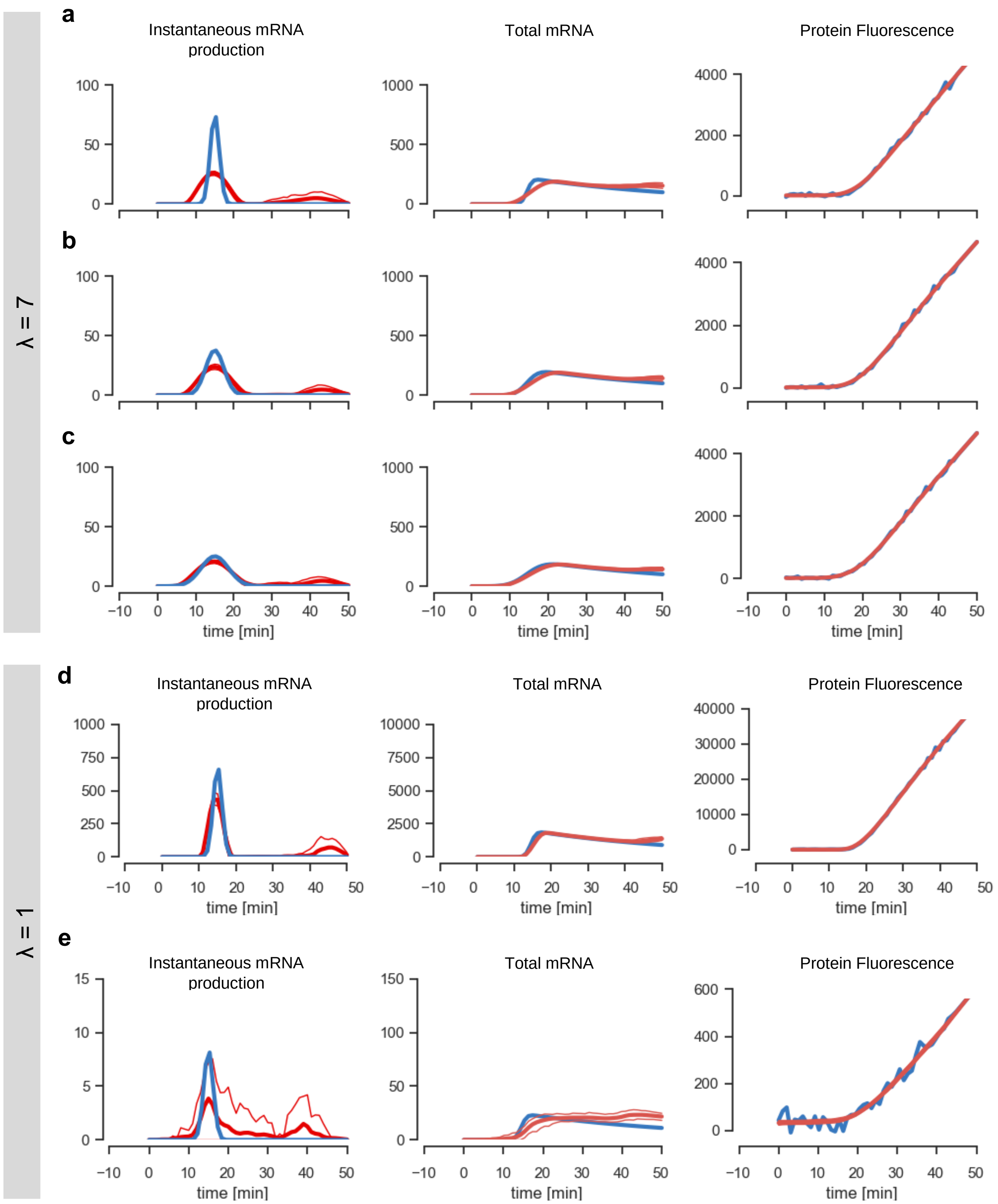


**Supplementary Figure 5. Inferring temporal resolution of mRNA reconstruction analysis with simulated data.** Protein fluorescence has been simulated starting from the time courses of mRNA production (leftmost column, in blue). We considered bursts of mRNA production with different durations producing the same total amount of

mRNA. The duration of the bursts (FWHM) is set to 5 minutes in panels **a**,**d** and **e**, 10 minutes in panel **b** and 15 min in panel **c**. Similar noise levels as those observed in our own experimental data for the Bcd3 enhancer have been added to the simulated fluorescence signal for panels **a**, **b** and **c** (rightmost column, in blue), whereas 10x weaker noise is added in panel **d** and 10x stronger noise in panel **e**. The mRNA reconstruction algorithm is used to reconstruct the total amount of mRNA and the rate of mRNA production rates by fitting the noisy fluorescence data. The regularization parameter λ is set to 7 in panels **a-c** and to 1 in panels **d** and **e**. The result of the reconstruction and corresponding confidence intervals are plotted in red in each panel. The high signal-to-noise ratio considered in panel **d** allows obtaining a robust reconstruction even at a setting of λ=1, which substantially improves temporal resolution. In contrast, at lower signal-to-noise ratios, such as those shown in panel **e**, it is not possible to obtain a robust reconstruction with λ=1; in this case the bootstrapping error of the instantaneous mRNA production rate becomes as large as the signal.

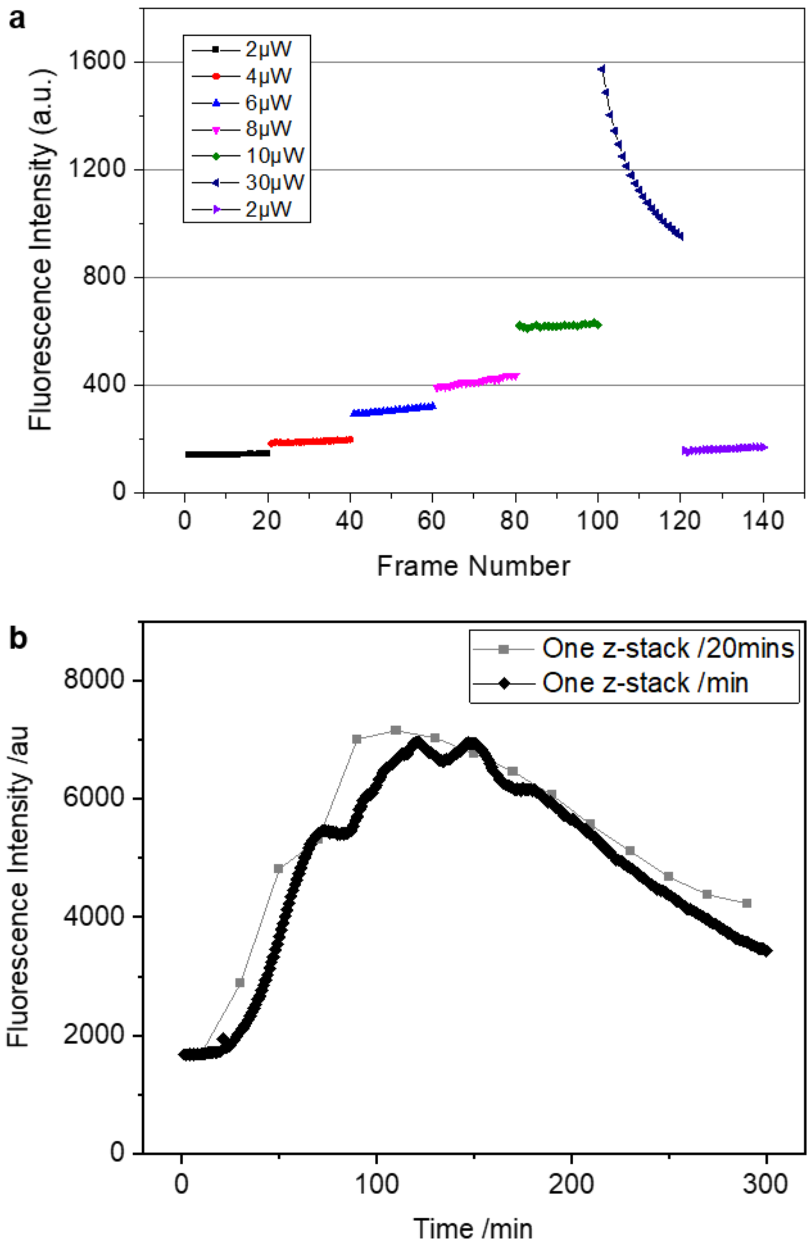


**Supplementary Figure 6**. **Optimization of the laser excitation power for the mNeon reporter sytem**. **a**. Average intensities of consecutive confocal fluorescence time series of the anterior part of a developping *hb_ant* embryo. The laser power was changed every 20 frames, in steps from 2µW to 30µW (measured at the entrance pupil of the objective) as indicated. The laser power was returned to 2µW for the the last cycle of confocal images to check that rate of expression of the mNeonGreen reporter increased only weakly during the measurements. The slope of the signal increases linearly with laser power until photobleaching becomes important. The optimal laser power is the one that generates the highest slope for the fluorescence

signal, while photodegradation is negligible. Here, the optimal laser power was found to be 8µW (pink points). **b**. Average fluorescence time course of two embryos carrying the *hb_ant* enhancer and imaged over 5 hrs with one frame per minute (our usual condition) and one frame every 20 mins, respectively. The fluorescence time courses of the two datasets overlap very well, demonstrating that photobleaching does not play a significant role. The shape of the curves is explained in more detail in **Supplementary Fig. 7**.

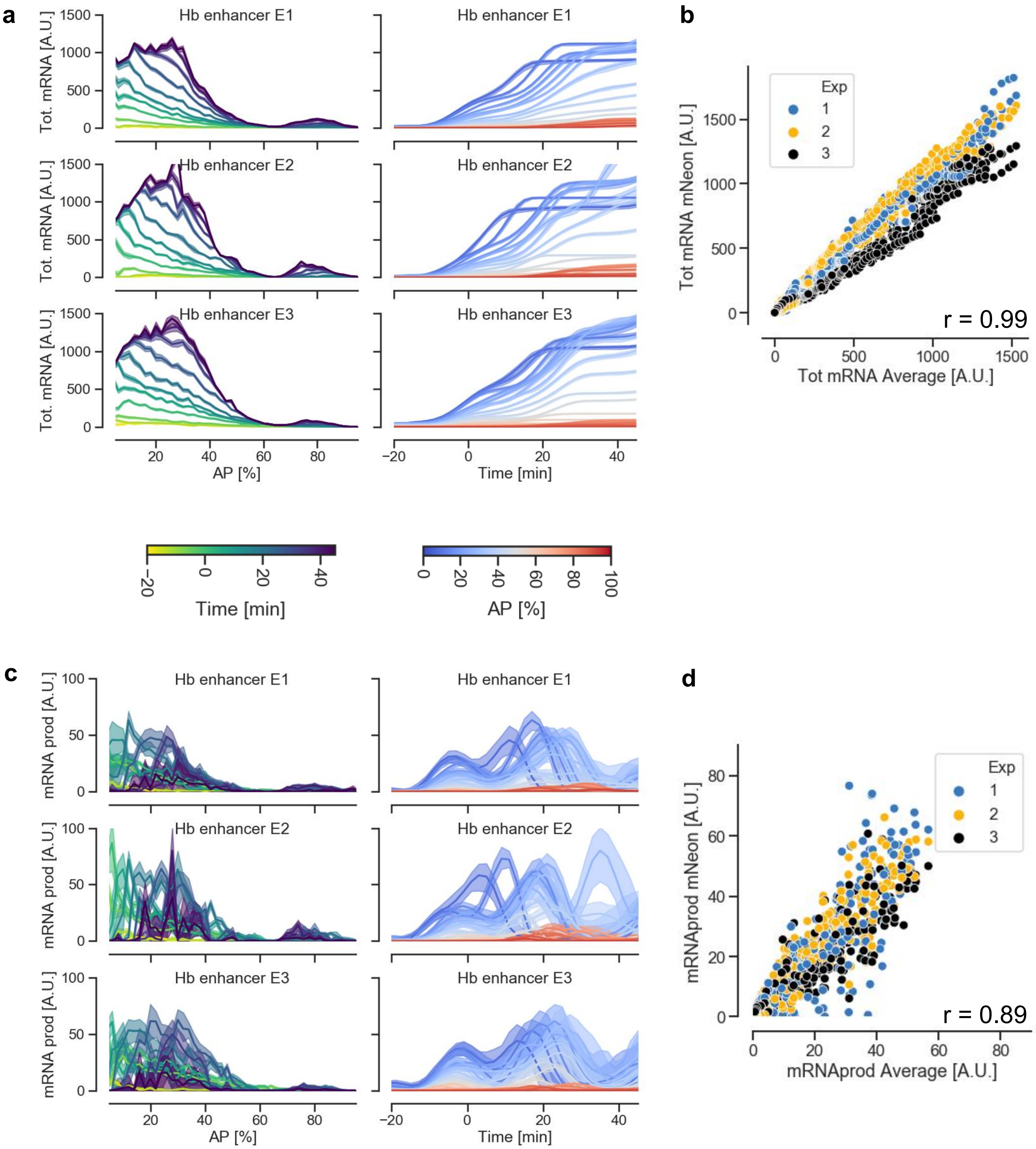


**Supplementary Figure 7. Reproducibility of mNeon reporter measurements for the hb_ant enhancer. a** Cumulative mRNA production patterns at different times of embryo development (left) and time course of cumulative mRNA production in 2% bins along the AP axis of the embryo (right) for three different embryos carrying the *hb_ant-*

*mNeonRep* construct. **b** Comparison of the three replicates for the measurement of the cumulative mRNA production, at all times and positions. **c** Pattern of the instantaneous mRNA production rate at different times of embryo development (left) and time course of mRNA production rate in 2% bins along the AP axis of the embryo (right) for three different embryos. **d** Comparison of the three replicates for the measurement of the mRNA production rate, at all times and positions.

**a**

***hb_ant* > mNeonRep mNeon Fluorescence signal**

| | |
|---|---|
| Stage 5 | 2h:10m-2h:50m |
| Stage 6 | 2h:50m-3h:00m |
| Stage 7-8 | 3h00m-3:40m |
| Stage 9-10 | 3h:40m-5h:20m |
| Stage 11 | 5h20m-7h20m |

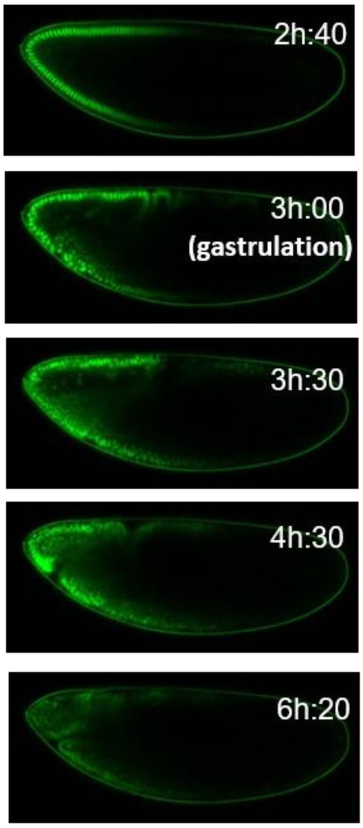


**b**

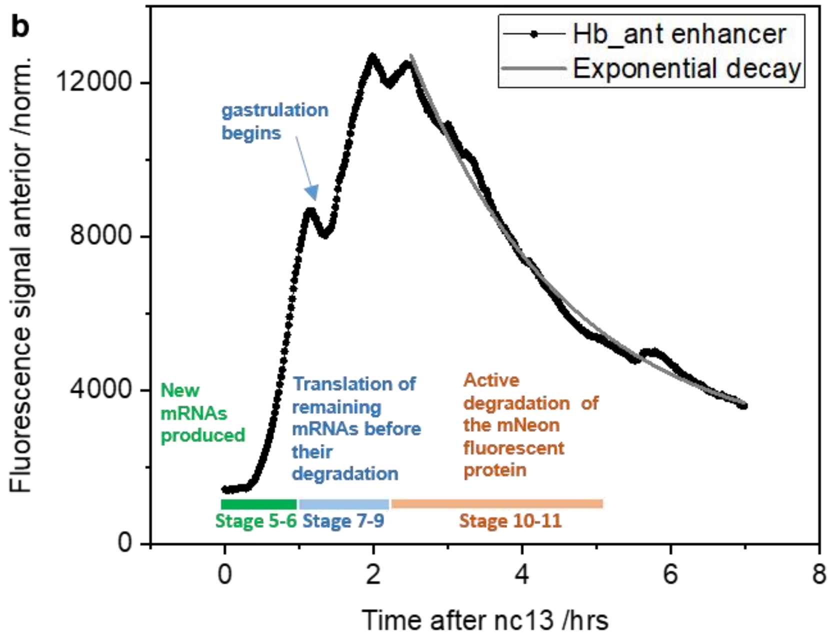


**Supplementary Figure 8. Reporter expression during later stages of embrogenesis**. **a** Confocal fluorescence images of mNeonRep for embryos carrying the *hb_ant* enhancer during embryonic stages 5-11 (time after fertilization indicated in the left panel). **b** Fluorescence time course of mNeonRep for the anterior part of the

embryo. Starting from stage 7 (corresponding to 50 mins after the onset of nc14, our time 0), the expression of the native hunchback locus, and thus presumably of the *hb_ant* enhancer, is largely shut down. However, we observe that the fluorescence reporter signal persists well into later stages. Following the strong increase of the fluorescence signal during stages 5-6 (highlighted in green), we see a more moderate increase during stages 7-9, which likely results from translation of previously produced mRNA molecules that are now progressively degraded (highlighted in blue). Finally, once no more mRNA molecules are present at stage 10, the fluorescence signal decreases exponentially due to active protein degradation. Note that due to the morphological changes and the rapid movement of nuclei that occur within the embryo starting with gastrulation, a more detailed quantitative analysis of this dynamic using our reconstruction algorithm is not possible. As shown in **Supplementary Fig. 5b**, the decrease of protein levels from stage 10 onwards is not caused by photobleaching, but is solely due to active protein degradation. Thus, we can use these data to extract an estimate of the *mNeonRep* protein lifetime, by fitting the fluorescence time course after stage 10 with an exponential decay function. We find a characteristic protein lifetime of ~130 mins.

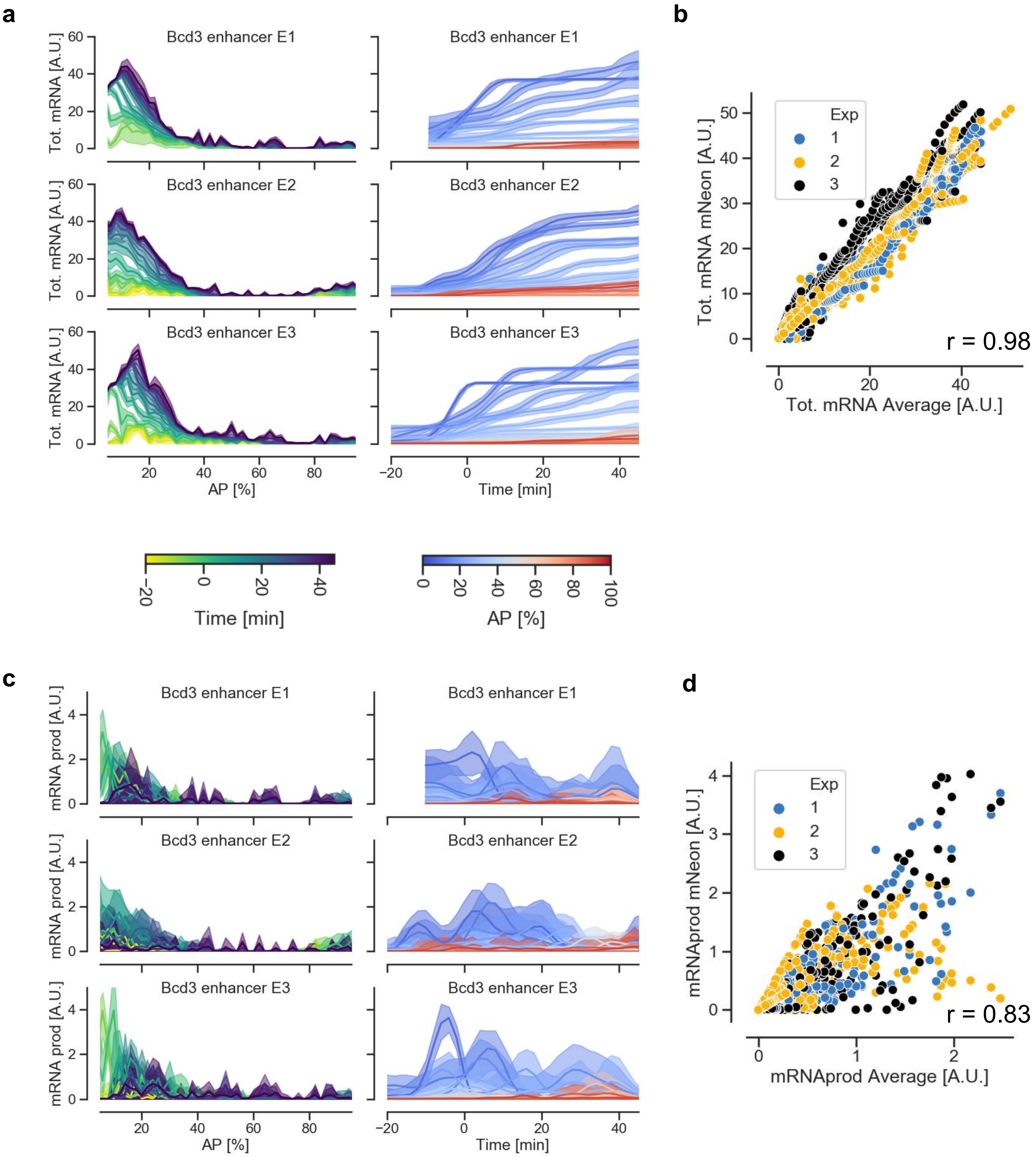
a
b
c
d
Bcd3 enhancer E1
Bcd3 enhancer E2
Bcd3 enhancer E3
Tot. mRNA [A.U.]
AP [%]
Time [min]
Tot. mRNA mNeon [A.U.]
Tot. mRNA Average [A.U.]
Exp
r = 0.98
mRNA prod [A.U.]
mRNAprod mNeon [A.U.]
mRNAprod Average [A.U.]
r = 0.83

**Supplementary Figure 9. Reproducibility of mNeon reporter measurements for the Bcd3 enhancer. a** Cumulative mRNA production patterns at different times of embryo development (left) and time course of cumulative mRNA production in 2% bins along the AP axis of the embryo (right) for three different embryos carrying the *Bcd3-mNeonRep* construct. **b** Comparison of the three replicates for the measurement of the cumulative mRNA production, at all times and positions. **c** Pattern of the instantaneous mRNA production rate at different times of embryo development (left) and time course of mRNA production rate in 2% bins along the AP axis of the embryo (right) for three different embryos. **d** Comparison of the three replicates for the measurement of the mRNA production rate, at all times and positions.

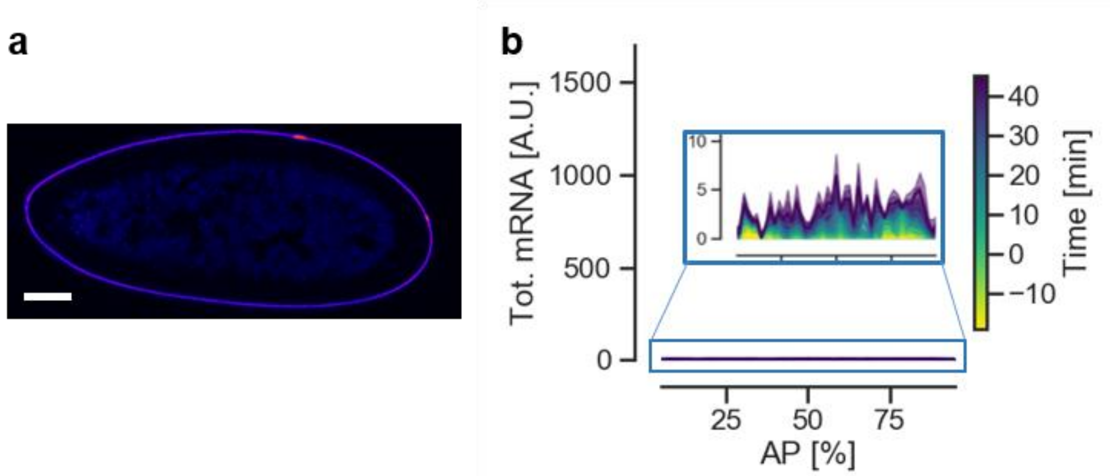


**Supplementary Figure 10. Imaging a wild type embryo as a negative control for the image and data analysis pipeline**. **a**. Confocal fluorescence section of a wild type embryo at the end of stage 5. While a weak autofluorescence can be observed in the vitelline membrane and in the yolk in the middle of the embryo, the background signal is extremely low in the cortical region of the embryo where the nuclei are, and where our measurements are performed. **b**. Inferred total mRNA levels along the AP axis of a wild-type embryo; the reconstruction was performed using the same data analysis pipeline that was applied to embryos expressing the mNeon reporter, including image segmentation, background correction and mRNA reconstruction. Note that the reconstruction does not show any obvious artefact or systematic error introduced by the analysis pipeline. Thus, this analysis allows to estimate the level of background noise of our reporter system: the noise amounts to ~5 A.U. of total mRNA, which is 10 times lower than the expression level of the weakest enhancer measured in this study (*Bcd3*).

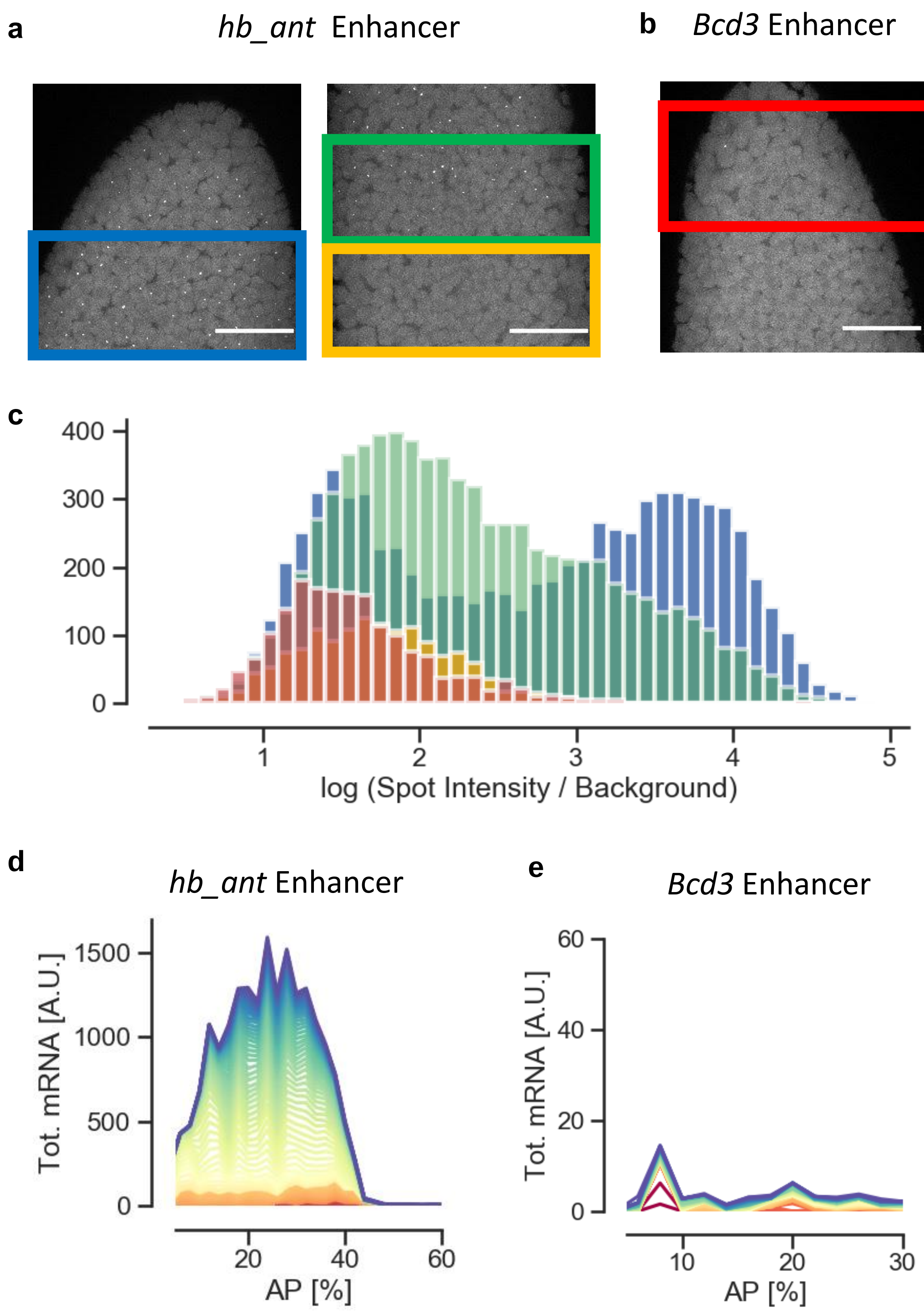


**Supplementary Figure 11. Transcriptional dynamics of the *hb_ant* and *Bcd3* enhancers measured with the MS2 reporter system.** **a** Snapshots of anterior and middle region of embryos expressing MS2 stem loops under the control of the *hb_ant* enhancer and **b** the *Bcd3* enhancer. **c** Histograms of the intensity of MS2-MCP fluorescent spots detected in different regions of embryos for the *hb_ant* and *Bcd3* enhancers. **d-e** Cumulative expression profiles at different times for the *hb_ant* and *Bcd3* enhancers, measured using the MS2-MCP system. The total mRNA at time t in

any bin along the AP axis has been defined as the sum over all previous times and over all nuclei present in a 2% EL window, divided by the width of the embryo at that position.

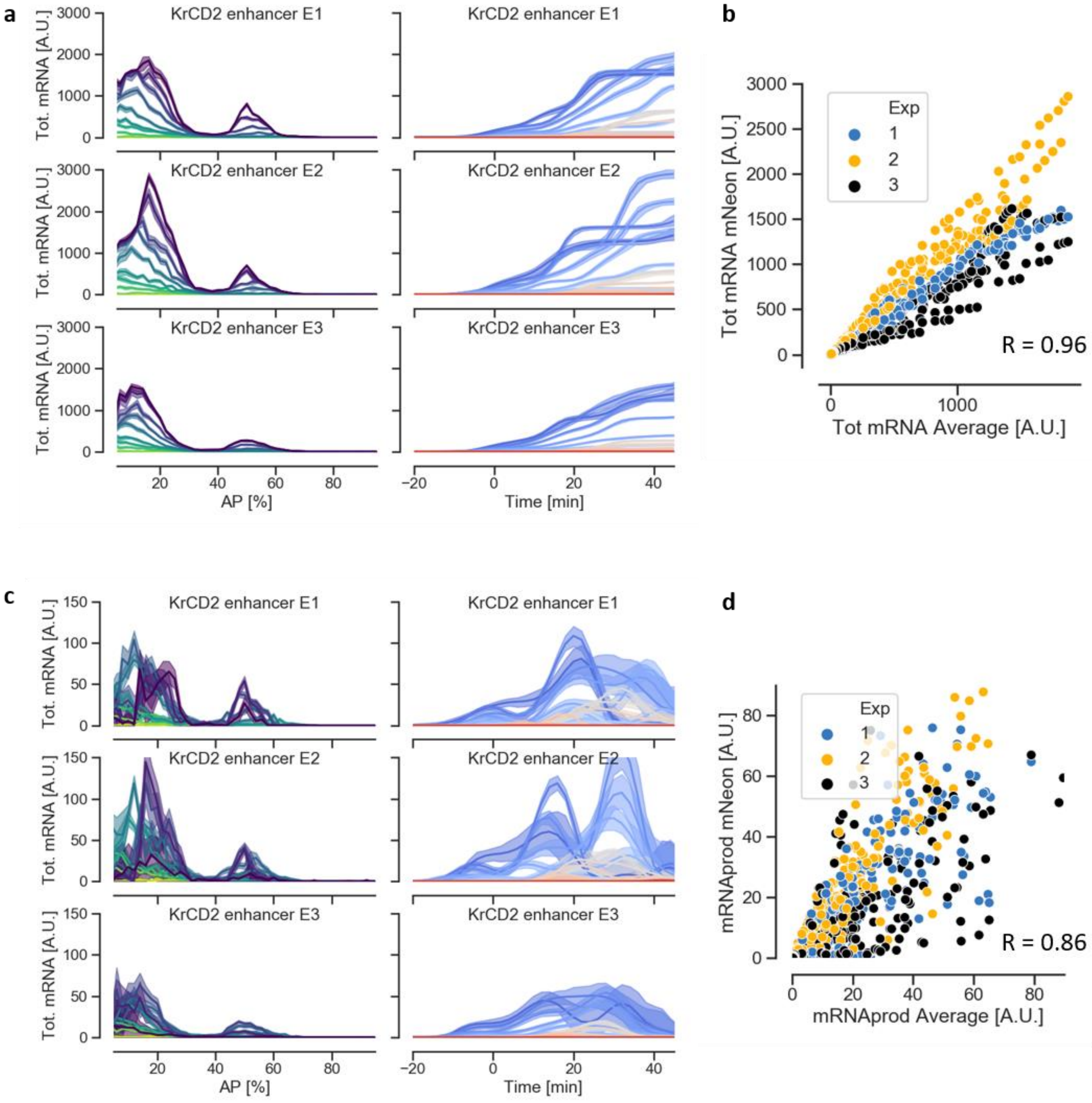
a
b
c
d
KrCD2 enhancer E1
KrCD2 enhancer E2
KrCD2 enhancer E3
Tot. mRNA [A.U.]
AP [%]
Time [min]
Tot mRNA mNeon [A.U.]
Tot mRNA Average [A.U.]
Exp
1
2
3
R = 0.96
mRNAprod mNeon [A.U.]
mRNAprod Average [A.U.]
R = 0.86

**Supplementary Figure 12: Reproducibility of mNeon reporter measurements for the *Kr_CD2* enhancer. a** Cumulative mRNA production patterns at different times of embryo development (left) and time course of cumulative mRNA production in 2% bins along the AP axis of the embryo (right) for three different embryos carrying the *Kr_CD2-mNeonRep* construct. **b** Comparison of the three replicates for the measurement of the cumulative mRNA production, at all times and positions. **c** Pattern of the instantaneous mRNA production rate at different times of embryo development (left) and time course of mRNA production rate in 2% bins along the AP axis of the embryo (right) for three different embryos. **d** Comparison of the three replicates for the measurement of the mRNA production rate, at all times and positions.

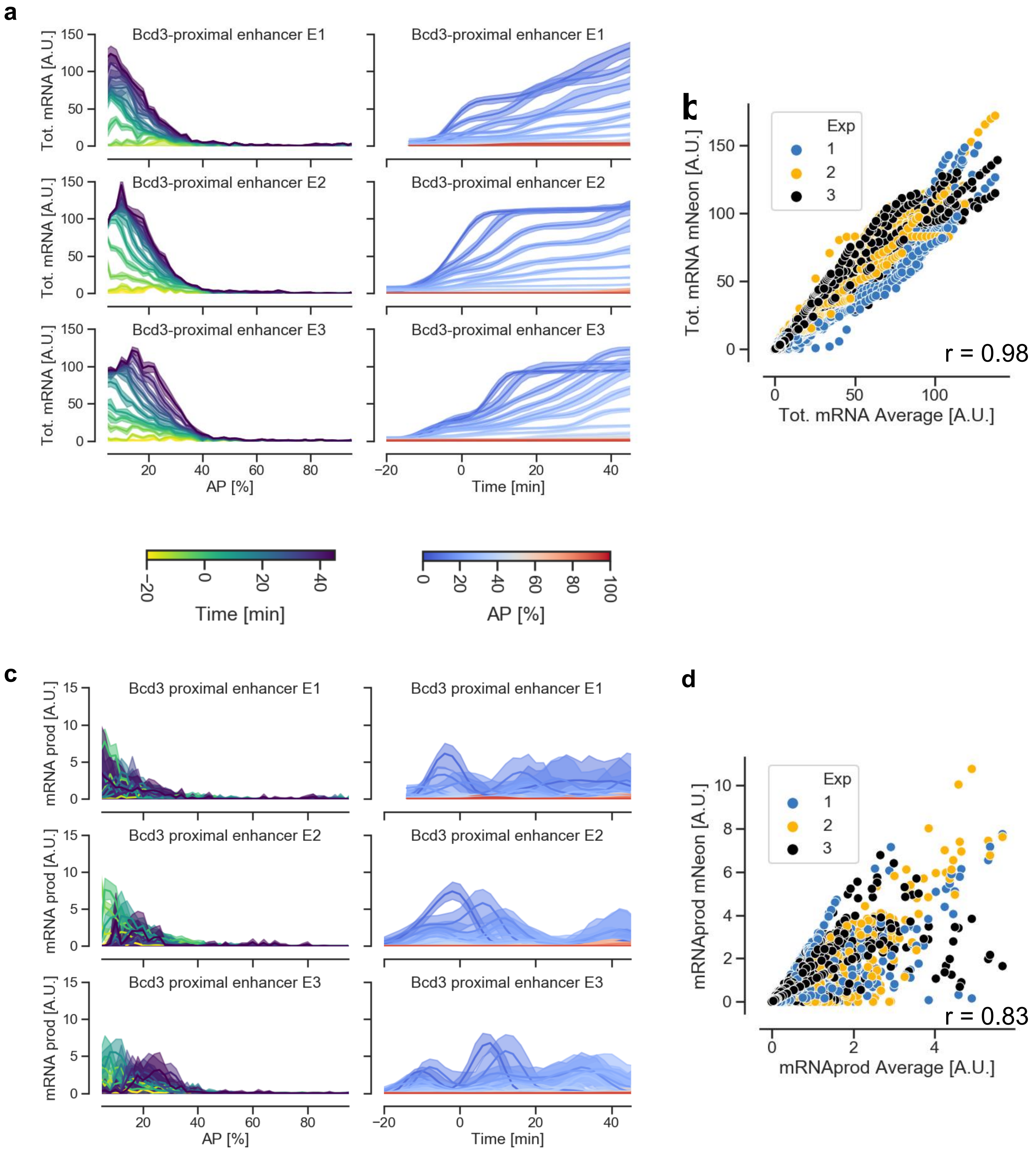


**Supplementary Figure 13. Reproducibility of mNeon reporter measurements for the *Bcd3-proximal* enhancer. a** Cumulative mRNA production patterns at different times of embryo development (left) and time course of cumulative mRNA production in 2% bins along the AP axis of the embryo (right) for three different embryos carrying

the *Bcd3proximal-mNeonRep* construct. **b** Comparison of the three replicates for the measurement of the cumulative mRNA production, at all times and positions. **c** Pattern of the instantaneous mRNA production rate at different times of embryo development (left) and time course of mRNA production rate in 2% bins along the AP axis of the embryo (right) for three different embryos. **d** Comparison of the three replicates for the measurement of the mRNA production rate, at all times and positions.

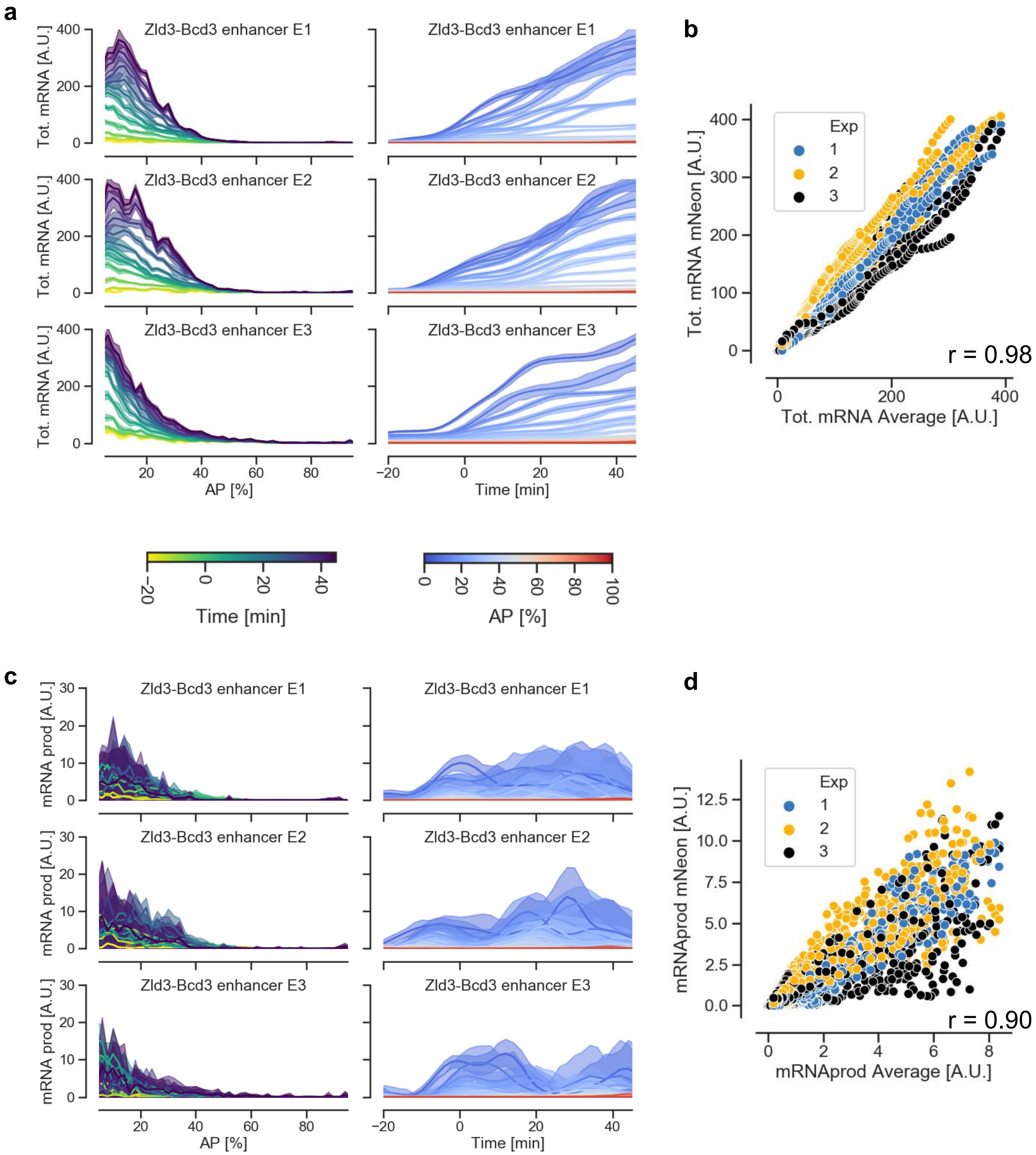


**Supplementary Figure 14. Reproducibility of mNeon reporter measurements for the *Zld3-Bcd3* enhancer. a** Cumulative mRNA production patterns at different times of embryo development (left) and time course of cumulative mRNA production in 2% bins along the AP axis of the embryo (right) for three different embryos carrying the *Zld3-Bcd3-mNeonRep* construct. **b** Comparison of the three replicates for the

measurement of the cumulative mRNA production, at all times and positions. **c** Pattern of the instantaneous mRNA production rate at different times of embryo development (left) and time course of mRNA production rate in 2% bins along the AP axis of the embryo (right) for three different embryos. **d** Comparison of the three replicates for the measurement of the mRNA production rate, at all times and positions.

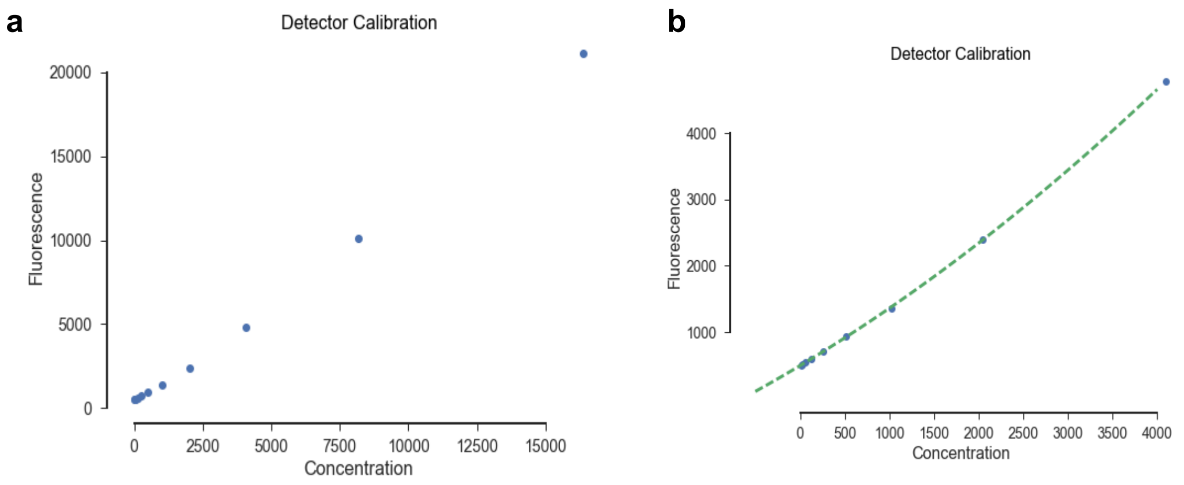


**Supplementary Figure 15. Linearity of the imaging setup**. **a** fluorescence signal from a serial dilution of Rhodamine6G in a 96 well plate as a function of dye concentration. **b** The result is slightly non-linear only for low dye concentrations and can been interpolated with a polynomial fit, which we used to correct the data obtained from expression measurements in living embryos.

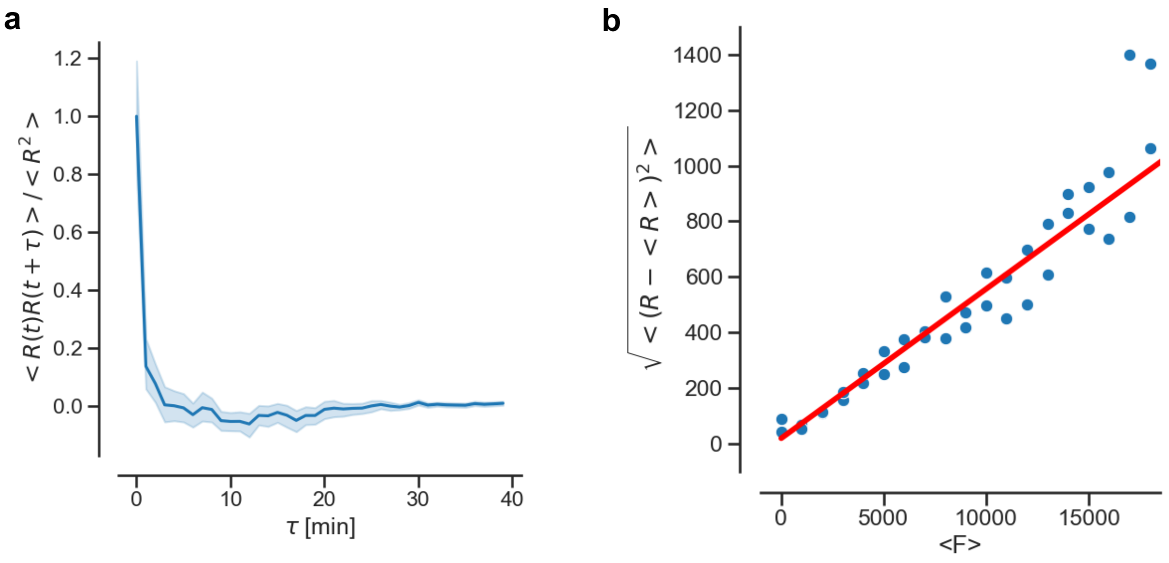


**Supplementary Figure 16. Statistical analysis of fit residuals. a** Autocorrelation function of the residuals of the mRNA reconstruction fitting procedure for *hb_ant* Enhancer. No time correlation of the residuals is observed. <> denotes the average of all the data at different positions and times. **b** The amplitude of the residuals of the mRNA reconstruction fitting procedure depends linearly on the intensity of the fluorescence signal.

## Construct Sequences and test of NLS arrangements

**mNeonReporter**

The mNeonGreen sequence has been fused C and N terminal to three different nuclear localization signals (NLS):

| | |
|---|---|
| Bipartite class, N-term NLS [1] | RKQVSSHIQVLARRKLR |
| Sv40-NLS, C-term – class1[2] | PKKKRKV |
| Class 3 NLS, C-term - strong[2] | AAAKRSWSMAF |

In order to determine the arrangement of NLSs that provides the strongest nuclear localization, various combinations of these motifs fused to mNeonGreen were tested through preliminary experiments in *Drosophila* S2 cells. To characterize the strength of the nuclear localization, the ratio of nuclear vs cytoplasmic fluorescence was used as a metric.

| | | | | | Nuc/Cyt Min | Nuc/Cyt Max |
|---|---|---|---|---|---|---|
| A | Bip | mNeonGreen | SV40 | - | 16 | 20 |
| B | Bip | mNeonGreen | - | - | 1 | 2 |
| C | Bip | mNeonGreen | SV40 | Class3 | 17.5 | 19.5 |
| D | - | mNeonGreen | SV40 | - | 2 | 3 |
| E | - | mNeonGreen | SV40 | Class3 | 2 | 6 |

All NLSs have been fused together and to mNeonGreen through short GS linker sequences. For the arrangement achieving the strongest localization (Construct C in the previous table) the resulting protein sequence reads:

MRKQVSSHIQVLARRKLRGSVSKGEEDNMASLPATHELHIFGSINGVDFDMVGQGT
GNPNDGYEELNLKSTKGDLQFSPWILVPHIGYGFHQYLPYPDGMSPFQAAMVDGS
GYQVHRTMQFEDGASLTVNYRYTYEGSHIKGEAQVKGTGFPADGPVMTNSLTAAD
WCRSKKTYPNDKTIISTFKWSYTTGNGKRYRSTARTTYTFAKPMAANYLKNQPMYV
FRKTELKHSKTELNFKEWQKAFTDVMGMDELYKGSPKKKRKVGSAAAKRSWSMA
F*

The corresponding coding sequence has been codon optimized for expression in *Drosophila Melanogaster* and results:

>mNeonRep

ATGCGGAAGCAAGTCTCGAGCCACATCCAGGTGTTGGCCCGACGCAAACTGCGTGGCTCCATGG
TGTCTAAGGGCGAAGAGGACAACATGGCGAGTCTACCAGCCACGCATGAGCTTCACATCTTTGG
CTCCATCAATGGAGTGGATTTCGATATGGTGGGTCAAGGAACTGGCAATCCGAATGACGGCTATG
AGGAACTGAACCTGAAGTCGACCAAGGGCGATTTGCAGTTTTCTCCCTGGATTCTGGTACCGCAT
ATTGGCTATGGCTTTCACCAGTACTTGCCATATCCTGATGGCATGTCACCTTTCCAAGCGGCAAT
GGTGGACGGTAGCGGCTATCAAGTCCATCGAACTATGCAGTTCGAGGATGGAGCCAGTCTGACC
GTGAACTACCGCTATACCTATGAAGGCTCCCACATTAAGGGTGAAGCTCAGGTGAAAGGAACAG
GCTTTCCAGCTGATGGTCCCGTTATGACGAACTCCTTGACTGCAGCCGACTGGTGCAGATCCAA
GAAAACCTACCCCAATGACAAGACCATCATCTCGACGTTCAAATGGTCGTATACGACAGGAAATG
GTAAGCGCTACCGTTCAACAGCGAGGACAACGTACACCTTCGCCAAACCGATGGCTGCCAACTA
CCTGAAGAACCAGCCCATGTACGTCTTTCGCAAGACCGAGCTCAAACACAGCAAGACTGAGCTG
AACTTCAAAGAGTGGCAGAAGGCCTTCACCGATGTTATGGGCATGGATGAGCTCTACAAGGGAT
CGCCGAAGAAGAAGCGGAAAGTCGGATCGGGCTCGGCTGCGGCCAAGCGCTCCTGGAGCATGG
CCTTC

## Enhancers

>Hb_ant

AACAATTGCAACAGGCATTAGTTTATATATCGCTCAGGTAGACGGATGCACGCGTCAAGGGATTA
GATGGGCAGAGGTGACGGGAAGTCAGGTACAGGTCGCGGATCGGTCGGGAATCGAGGATCACG
GATCGCGGATTGAGGATTGCGCTCTTGATCCATTCTGGATTAGAGCAGAAACAAAAAATTATGCG
CACTTGGATTTGGATGATCCGGGAGCTTAGCGGATGGCCAGCTTAGCAGCGAGCTGCGAATTTT
CCACCGGTTTTCTATGGGGATTACGTTGGTCAGGAGTCGACAGCAGGAGTAGGCAGCTAGCGTG
GGCAGTTTCGTAGTTAATAATAAAAAGTAAAAAGGATTGCGGGACTTAACTAAATTAACGGATCAG
AACTGCTTACACCTGCGGGAAAACTCTAAGGACCAACTAAACTATATGCATAATATGTGCAGTATA
ATTATTACACACCCATTTGAAAAACATTTTCCTGACAACAATTTTCCGCCAGACATTTCACTTTGAT
TTGCGTAGTTTTTCTAATAATTCTCGCATTAAAATTGCTTGTTGCCTATATTTTTTCCATTTCCAATT
TCACACTGAAAAATTGTGCAGTTGCTGCATTTTTGGCTAATTGTTTGTGCTTTCAAGTAAATATTAT
TAAAAACGCAAAACGGGAAAAAGGGGCATTTACGGAATATTATTATGGGAGGATGGTGCTGTGCT
A

>Bcd3

AGGTTCTAATCCCGGTCTAATCCCTCGAGTCTAATCCCATGAGTCGAC

>Zld3-Bcd3

CGGAAGTTCAGGTATTGCTATTCAGGTAGAGGCCGTACGTGCAGGTAACCTTGCGTAGGTTCTAA
TCCCGGTCTAATCCCTCGAGTCTAATCCCATGAGTCGACG

## Linkers

Enhancer-promoter linker sequence, removed in the Bcd3-proximal enhancer:

>Linker

AGGTTCCAGTGTGGTGGAATTCTGCAGATATCCAGCACAGTGGCGGCCGCTCGAGTCTAGAGGG
CCCTTCGAA

Spacer sequence, inserted upstream the enhancer sequence to create a neutral background around enhancers:

>Spacer

GAATTCGGCCGGCCCACCAAACCGCTTTAGTCCCGCCAGCCAGCCACCAGTGGTGCAGCCCAC
CTCCTCGGCCCGCCAGTCGCCCATCGATGTCTGCCTGGAGGAGGATGTTCACTCCGTGCACAGC
CATCAGTCGTCCGCAAGCCTCCTGCATCCCATTGCCATCCGAGCCACGCCAACCACTCCGACTA
GCAGCAGCCCGCTGAGTTTTGCGGCCAAGATGCAGAGCTTGTCGCCCGTTTCGGTTTGCTCCAT
TGGCGGCGAAACCACCAGCGTTGTACCAGTGCATCCTCCCACCGTTTCCGCTCAAGAAGGACCC
ATGGATCTGAGCATGAAGACCTCGCGGAGCTCCGT

## REFERENCES


1. Magico, A.C. & Bell, J.B. Identification of a classical bipartite nuclear localization signal in the Drosophila TEA/ATTS protein scalloped. *PLoS One* **6**, e21431 (2011).
2. Kosugi, S. et al. Six classes of nuclear localization signals specific to different binding grooves of importin alpha. *J Biol Chem* **284**, 478-485 (2009).
