# Supplementary material for: A sensitive mNeonGreen reporter system to measure transcriptional dynamics in Drosophila development
Source: Commun Biol. 2020 Nov 12;3:663. doi: 10.1038/s42003-020-01375-5 (PMC7665215; doi:10.1038/s42003-020-01375-5)
Supplement: Supplementary file 3 — Description of Additional Supplementary Files [file 42003_2020_1375_MOESM3_ESM.pdf]

Description of Additional Supplementary Files

File Name: Supplementary Data 1
Description: Raw fluorescence intensities time courses and the extracted mRNA levels for the natural enhancers (Hb_ant and Kr_CD2).

File Name: Supplementary Data 2
Description: Extracted fluorescence intensities obtained with the MS2-MCP system for the Hb_ant and the Bcd3 enhancers.

File Name: Supplementary Data 3
Description: Raw fluorescence intensities time courses and the extracted mRNA levels for the natural enhancers (Bcd3-proximal, Bcd 3, and Zld-Bcd3).

File Name: Supplementary Movie 1
Description: Spatiotemporal activity of the *hb_ant* enhancer imaged by confocal microscopy (maximum intensity projection) and using the sensitive mNeonGreen reporter system.

File Name: Supplementary Movie 2
Description: Spatiotemporal activity of the hb_ant enhancer imaged by confocal microscopy (maximum intensity projection) and using the MS2-MCP reporter system. The typical MS2-MCP fluorescent spots can be observed in the anterior half of the embryo.

File Name: Supplementary Movie 3
Description: Spatiotemporal activity of the *Bcd3* enhancer imaged by confocal microscopy (maximum intensity projection) and using the sensitive mNeonGreen reporter system. we detect a weak gradient of reporter expression that slowly decreases.

File Name: Supplementary Movie 4
Description: Spatiotemporal activity of the *Bcd3* enhancer imaged by confocal microscopy (maximum intensity projection) and using the MS2-MCP reporter system. A limited number of very weak fluorescents dots are almost indistinguishable from the background noise.

File Name: Supplementary Movie 5
Description: Spatiotemporal activity of the *Kr_CD2* enhancer imaged by confocal microscopy (maximum intensity projection) and using the sensitive mNeonGreen reporter system.
