# Supplementary material for: A sensitive mNeonGreen reporter system to measure transcriptional dynamics in Drosophila development
Source: Commun Biol. 2020 Nov 12;3:663. doi: 10.1038/s42003-020-01375-5 (PMC7665215; doi:10.1038/s42003-020-01375-5)
Supplement: Supplementary file 12 — Reporting Summary [file 42003_2020_1375_MOESM12_ESM.pdf]

nature research

Corresponding author(s): Christophe Jung

Last updated by author(s): Sep 21, 2020

Reporting Summary

Nature Research wishes to improve the reproducibility of the work that we publish. This form provides structure for consistency and transparency in reporting. For further information on Nature Research policies, see our Editorial Policies and the Editorial Policy Checklist.

Statistics

For all statistical analyses, confirm that the following items are present in the figure legend, table legend, main text, or Methods section.

n/a	Confirmed	
☐	☒	The exact sample size (*n*) for each experimental group/condition, given as a discrete number and unit of measurement
☐	☒	A statement on whether measurements were taken from distinct samples or whether the same sample was measured repeatedly
☐	☒	The statistical test(s) used AND whether they are one- or two-sided<br>*Only common tests should be described solely by name; describe more complex techniques in the Methods section.*
☒	☐	A description of all covariates tested
☒	☐	A description of any assumptions or corrections, such as tests of normality and adjustment for multiple comparisons
☐	☒	A full description of the statistical parameters including central tendency (e.g. means) or other basic estimates (e.g. regression coefficient) AND variation (e.g. standard deviation) or associated estimates of uncertainty (e.g. confidence intervals)
☒	☐	For null hypothesis testing, the test statistic (e.g. *F*, *t*, *r*) with confidence intervals, effect sizes, degrees of freedom and *P* value noted<br>*Give P values as exact values whenever suitable.*
☒	☐	For Bayesian analysis, information on the choice of priors and Markov chain Monte Carlo settings
☒	☐	For hierarchical and complex designs, identification of the appropriate level for tests and full reporting of outcomes
☒	☐	Estimates of effect sizes (e.g. Cohen's *d*, Pearson's *r*), indicating how they were calculated

Our web collection on statistics for biologists contains articles on many of the points above.

Software and code

Policy information about availability of computer code

Data collection	image acquisition has been performed with Zeiss ZEN (2009)
Data analysis	Image analysis has been performed with the Definiens XD 2.0 software package (Munich, Germany) and data have been further analyzed with custom Python3 scripts (details are provided in the Methods section).

For manuscripts utilizing custom algorithms or software that are central to the research but not yet described in published literature, software must be made available to editors and reviewers. We strongly encourage code deposition in a community repository (e.g. GitHub). See the Nature Research guidelines for submitting code & software for further information.

Data

Policy information about availability of data

All manuscripts must include a data availability statement. This statement should provide the following information, where applicable:

- Accession codes, unique identifiers, or web links for publicly available datasets
- A list of figures that have associated raw data
- A description of any restrictions on data availability

Raw data associated with all figures are available as Supplementary Data 1, 2 and 3. There is no restriction about data availibility.

Field-specific reporting

Life sciences study design

All studies must disclose on these points even when the disclosure is negative.

Sample size	For every enhancer-reporter construct in this study, three or more biological replicates were measured.
Data exclusions	We discarded only movies affected by technical issues, e.g. in which the sample moved during the experiment, making the analysis more difficult or not possible. After this exclusion, we could always analyze three independent biological replicates for each enhancer-reporter construct.
Replication	All attempts of replication were successful.
Randomization	Organisms have been grouped based on the carried enhancer-reporter construct.
Blinding	Blinding was not relevant in this study.

Reporting for specific materials, systems and methods

We require information from authors about some types of materials, experimental systems and methods used in many studies. Here, indicate whether each material, system or method listed is relevant to your study. If you are not sure if a list item applies to your research, read the appropriate section before selecting a response.

Materials & experimental systems

n/a	Involved in the study
☒	☐ Antibodies
☒	☐ Eukaryotic cell lines
☒	☐ Palaeontology and archaeology
☐	☒ Animals and other organisms
☒	☐ Human research participants
☒	☐ Clinical data
☒	☐ Dual use research of concern

Methods

n/a	Involved in the study
☒	☐ ChIP-seq
☒	☐ Flow cytometry
☒	☐ MRI-based neuroimaging

Animals and other organisms

Policy information about studies involving animals; ARRIVE guidelines recommended for reporting animal research

Laboratory animals	Transgenic Drosophila Melanogaster fly stocks were generated by integrating reporter constructs in the same attP2 docking site using PhiC31 integrase.
Wild animals	n.a.
Field-collected samples	n.a.
Ethics oversight	No ethical approval was required for this study.

Note that full information on the approval of the study protocol must also be provided in the manuscript.
